# Aptamer-Based Label-Free Colorimetric Assay Using Gold Nanoparticles for Specific Detection of *Streptococcus suis*

**DOI:** 10.3390/bios16040215

**Published:** 2026-04-10

**Authors:** Sirikwan Sangboonruang, Natthawat Semakul, Apinyapat Matchawong, Anuchit Sattaphan, Kanokwan Saengsawang, Chatchawan Srisawat, Khajornsak Tragoolpua, Chayada Sitthidet Tharinjaroen

**Affiliations:** 1 Office of Research Administration, Chiang Mai University, Chiang Mai 50200, Thailand; sirikwan.sang@cmu.ac.th; 2 Division of Clinical Microbiology, Department of Medical Technology, Faculty of Associated Medical Sciences, Chiang Mai University, Chiang Mai 50200, Thailand; apinyapat.mat@cmu.ac.th (A.M.); sattaphan.ac@gmail.com (A.S.); khajornsak.tr@cmu.ac.th (K.T.); 3 Department of Chemistry, Faculty of Science, Chiang Mai University, Chiang Mai 50200, Thailand; natthawat.semakul@cmu.ac.th; 4 Department of Medical Technology, Lampang Hospital, Lampang 52000, Thailand; eds_672@hotmail.com; 5 Department of Biochemistry, Faculty of Medicine Siriraj Hospital, Mahidol University, Bangkok 10700, Thailand; chatchawan.sri@mahidol.ac.th; 6 Infectious Diseases Research Unit (IDRU), Faculty of Associated Medical Sciences, Chiang Mai University, Chiang Mai 50200, Thailand

**Keywords:** *Streptococcus suis*, aptamer, gold nanoparticles, aptasensor, colorimetric detection

## Abstract

*Streptococcus suis* is a serious zoonotic pathogen responsible for rapid progression and deadly infections in both humans and pigs. With an increasing number of reported cases and considering the limitations of standard routine identification, a simple, rapid, and cost-effective approach is needed. In this study, a label-free colorimetric assay based on gold nanoparticles (AuNPs) was applied with a specific aptamer, R8-su12. This assay offered simplified detection through observable color change, enabling visual analysis by the naked eye or assessment via UV–Vis spectrophotometry. Under the optimal assay conditions, the detection procedure was carried out within 45 min. The reaction of the aptasensor and other bacterial species, including *Staphylococcus aureus*, *S. pneumoniae*, *S. pyogenes*, *Pseudomonas aeruginosa*, *Escherichia coli*, *Enterococcus faecium*, and *E. faecalis*, was not present, indicating the specificity of this assay. Moreover, the aptasensor exhibited high sensitivity with a limit of detection (LOD) at 1 CFU of *S. suis* and had broad reactivity with *S. suis* serotypes 1, 1/2, 9, and 14, as well as with *S. suis* isolated from clinical specimens. Thus, this aptasensor demonstrates proof-of-concept feasibility including clinical sample testing before practical implementation. It holds promise as a practical tool for the early screening and outbreak management of *S. suis* in a variety of settings, such as clinical laboratories, food safety, and the environment.

## 1. Introduction

*Streptococcus suis* (*S. suis*) is a major zoonotic pathogen that primarily affects pigs but also poses serious risks to human health. It can cause a range of severe illnesses, including meningitis, septicemia, and toxic shock syndrome, often progressing rapidly with high fatality rates in critical cases. Human infection typically occurs through direct or indirect contact with infected pigs or by consuming contaminated pork products. Traditional bacterial culture methods remain widely used for *S. suis* detection, though they are limited by low sensitivity and long turnaround times [1]. Somehow, it also showed controversial results, leading to misdiagnosis and a delay in treatment.

Aptamers, short single-stranded DNA or RNA molecules, can bind to specific targets with high affinity and specificity. Generated through the Systematic Evolution of Ligands by Exponential Enrichment (SELEX) process, they form unique three-dimensional structures that enhance binding performance. Compared to traditional antibodies, aptamers offer several advantages: they are more stable, less immunogenic, and cheaper to produce. These properties make them valuable in diagnostics, therapeutics, and biosensing applications [2,3]. Recently, highly specific aptamers targeting *S. suis* have been developed, demonstrating strong potential for application in aptasensor-based detection systems [4]. In the last decade, aptamers have been utilized as specific recognition probes in several detection platforms, such as electrochemical and colorimetric sensors. As a simple, rapid, and low-cost detection method, the colorimetric assay has been widely applied to various routine analyses [5,6].

Gold nanoparticles (AuNPs) serve as an effective colorimetric indicator due to their surface plasmon resonance (SPR), causing an optical absorption spectrum. The alteration of the absorption spectrum depends on the particle size and aggregation state of AuNPs. The maximum optical absorption of AuNPs is approximately 520 nm in the monodisperse state, whereas the aggregated AuNPs increase the size of particles, resulting in a visible color shift from red to purple or blue [7]. Thus, the colorimetric biosensors based on aptamer-AuNPs can be developed as a useful tool for rapid screening tests for *S. suis*.

In this study, the colorimetric detection system of *S. suis* was established, using the R8-su12 aptamer (R8-su12 Apt) as a specific recognition probe and AuNPs as a colorimetric indicator. The assay parameters, such as NaCl concentration, aptamer concentration, and binding time, were optimized, and the color change of the AuNPs was monitored with the naked eye and spectroscopy. The specificity, limit of detection (LOD), and broad serotype reactivity between different serotypes were examined. Moreover, the isolates from clinical samples were used to evaluate the efficacy of the developed assay. These results demonstrate the feasibility of this colorimetric aptasensor as a simple, rapid, and sensitive screening method for *S. suis*. Therefore, further design and development are needed to make it usable as a rapid screening tool or a field setting device.

## 2. Materials and Methods

### 2.1. Materials and Reagents

HAuCl_4_ and trisodium citrate dihydrate were purchased from Sigma-Aldrich (Sigma-Aldrich, St. Louis, MO, USA) and RCI Labscan Ltd. (RCI Labscan CO., Ltd., Bangkok, Thailand), respectively. The reagents were of analytical grade. R8-su12 Apt was obtained according to our previous study [4], with some modification; all cytosine (C) and uracil (U) residues were 2′-fluoro-modified; (5′ CAU ACU GAG UAA GAU CGG AAA UUU CGG UUU AAG GCC ACG G 3′) was synthesized by GenePharma (Shanghai GenePharma Co., Ltd., Shanghai, China).

### 2.2. Bacterial Strains and Culture

*S. suis* and other bacteria, namely, *S. aureus* ATCC 25923, *S. pneumoniae* ATCC 49619, *S. pyogenes* ATCC 19615, *P. aeruginosa* ATCC 27853, *E. coli* ATCC 25922, *E. faecium* ATCC 4743, and *E. faecalis* ATCC 4736 (obtained from the Division of Clinical Microbiology, Faculty of Associated Medical Sciences, Chiang Mai University) and five clinical *S. suis* isolates (obtained from the Department of Medical Technology, Lampang Hospital), were inoculated on blood agar and incubated in a 5% CO_2_ incubator (Thermo Fisher Scientific Inc., Waltham, MA, USA) at 37 °C for 18–24 h. To prepare the bacterial suspension for the experiment, after culturing, all bacteria were harvested and washed three times with phosphate-buffered saline (PBS). Next, the bacterial suspension was adjusted to an optical density at 600 nm of 0.35, corresponding to 10^8^ CFU/mL. These bacterial numbers were confirmed by standard plate counting on blood agar (incubated at 37 °C, 5% CO_2_, 18–24 h) in three independent experiments. Then, the bacterial cells were diluted 10-fold to get the desired concentration.

The protocol was reviewed and approved by the Institutional Biosafety Committee, Chiang Mai University (permit number: CMUIBC A-0565014).

### 2.3. AuNPs Synthesis

The AuNPs were synthesized by the sodium citrate reduction of HAuCl_4_ with some modification [8]. Briefly, a 100 mL boiling solution of HAuCl_4_ (1 mM) was prepared under vigorous stirring. Then, 10 mL of sodium citrate solution (38.8 mM) was rapidly injected, and boiling continued for 15 min while stirring, which resulted in a color change of the mixture from pale yellow to a reddish wine-like color, indicating nanoparticle formation. The solution was stirred for another 10 min, and the resulting wine-red solution was cooled to room temperature (RT). The particle size and zeta potential (ZP) of AuNPs were measured using dynamic light scattering (DLS) with Zetasizer (Malvern Instruments Ltd., Malvern, Worcestershire, UK). Moreover, 10 μL droplets of the sample were dropped onto a piece of a carbon-coated copper grid and left to air-dry for visualization using scanning transmission electron microscopy (STEM). The STEM images were acquired using a JSM-IT800 Ultrahigh Resolution Field Emission SEM (JEOL, Peabody, MA, USA). UV–visible spectroscopy (Agilent BioTek, Winooski, VT, USA) was performed, and the spectral analysis was carried out at wavelengths between 400 and 700 nm. Characteristics of the AuNPs were described in Supporting Information (Figure S1). The reliability and reproducibility of the synthesized AuNPs were rigorously characterized across two independent batches, with a degree of consistency (inter-batch relative standard deviation (RSD)) lower than 5%, as described in Supporting Information (Figure S1 and Table S1).

### 2.4. Optimization of Colorimetric Assay

To develop the colorimetric assay, the optimal conditions, including NaCl concentration, aptamer concentration, and binding time, were investigated. The effect of NaCl in terms of inducing AuNP aggregation was determined with different concentrations of NaCl solution. The AuNP solution (100 μL) was mixed with PBS (10 μL) and NaCl, respectively, to obtain the final concentrations of NaCl at 0, 10, 20, 40, 80, and 100 mM, with the total volume of the mixture standing at 200 μL. The results were observed by visual inspection and by measuring the absorbance at the wavelength between 400 and 700 nm. The disperse phase is indicated by the maximum absorption intensity at 520 nm, and an increased absorption peak at 630 nm implies the aggregation of AuNPs. The ratio of absorption values at 630 nm and 520 nm (A630/520) was determined to assess the sensing reaction compared to the control [9]. The minimum concentration of NaCl that triggered aggregation, with color changing from red to purple, was selected for further analysis.

The optimization of aptamer concentration was then performed. Firstly, the aptamer was folded by incubating at 95 °C for 5 min and kept at RT for another 5 min. The colorimetric analysis was carried out as follows: 100 μL AuNPs and 10 μL of aptamer (0, 25, 50, 75, and 100 nM) were added to 96-well microtiter plates for 15 min. Then, 10 μL of PBS was added and incubated for 20 min. The NaCl solution was subsequently added with 200 μL total volume of the mixture. The reactions were observed by visual inspection and by measuring absorbance at wavelengths between 400 and 700 nm. Then, the ratio of A630/520 was determined. The optimal concentration of the aptamer for protecting the AuNPs from salt-induced aggregation was chosen.

For the determination of binding time, the assay was performed as mentioned earlier. Briefly, after the mixing of AuNPs and aptamer for 15 min, 10 μL of *S. suis* suspension in PBS at 10^3^, 10^4^, and 10^5^ CFU was added and incubated with different binding times (20, 30, and 40 min). After the addition of NaCl, the color change in the AuNP solution was observed, and the SPR spectrum was measured as previously described.

### 2.5. Specificity Testing of the Colorimetric Aptasensor

To examine the specificity of the assay, other bacterial strains, including *S. aureus* ATCC 25923, *S. pneumoniae* ATCC 49619, *S. pyogenes* ATCC 19615, *P. aeruginosa* ATCC 27853, *E. coli* ATCC 25922, *E. faecium* ATCC 4743, and *E. faecalis* ATCC 4736, were tested. The bacterial cultures were grown on blood agar for 24 h. The isolated colonies were suspended in PBS and adjusted to 10^5^, 10^6^, and 10^7^ CFU/mL. Then, 10 μL of each strain was tested directly with the developed assay, as previously mentioned.

### 2.6. Evaluation of the Colorimetric Aptasensor

#### 2.6.1. Limit of Detection (LOD)

To evaluate the LOD of the assay, *S. suis* serotype 2 reference strain P1/7 (S2 P1/7) was cultured, and bacterial numbers were adjusted. Then, various bacterial cell numbers (10^0^–10^6^ CFU) were tested with the assay. The result was further interpreted in three independent experiments using the A630/A520 ratio.

#### 2.6.2. Detection of *S. suis* Non-Serotype 2 P1/7 Isolates and *S. suis* Derived from Clinical Samples

To test the broad serotype reactivity of the assay with other *S. suis* serotypes, serotypes 1 (S1), 1/2 (S1/2), 2 (S2), 9 (S9), and 14 (S14) were prepared. In addition, 5 isolates of *S. suis* derived from clinical specimens were tested to support the efficiency of the developed assay.

### 2.7. Statistical Analysis

All results were performed in three independent replicates, and the results are presented as mean ± standard error of the mean (SEM). Comparisons between groups were made using one-way analysis of variance (ANOVA) and Dunnett’s multiple comparisons test using GraphPad Prism version 9.0 (GraphPad Software Inc., San Diego, CA, USA). Notably, * *p* < 0.05, ** *p* < 0.01, and *** *p* < 0.001 were statistically significant.

## 3. Results

### 3.1. Principle of the Colorimetric Aptasensor

**Figure 1:**
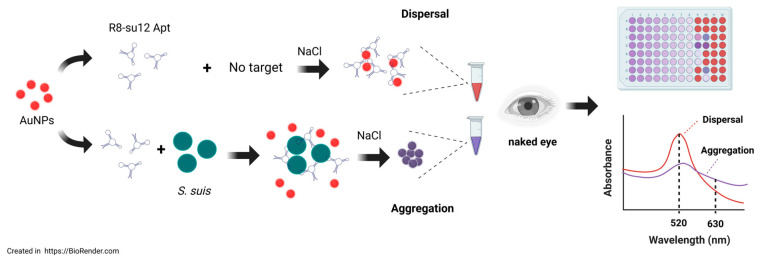
Principle of colorimetric aptasensor based on AuNPs for *S. suis* detection. The figure was created in https://BioRender.com. Access date 26 February 2026.

The mechanism of the colorimetric aptasensor, used for detecting *S. suis* based on AuNPs, is illustrated in Figure 1. In the dispersed phase, the aqueous solution of AuNPs presents a red color. Upon the addition of NaCl, the aggregation of AuNPs occurs and results in a color change from red to purple-gray. In the presence of the aptamer, the nitrogen bases of the aptamer can attach to the AuNP surface by a non-covalent bond. As a huge negative electricity group, the aptamer stabilizes AuNPs through electrostatic repulsion and prevents salt-induced aggregation. Therefore, the solution remains red. When *S. suis* exists in the sample, the aptamer specifically binds to the bacterial targets and detaches from the AuNP surface. Without the protective aptamer, AuNPs are exposed to NaCl, resulting in the aggregation state with the color changing from red to purple-gray.

### 3.2. Characterization of the Colorimetric Aptasensor

**Figure 2:**
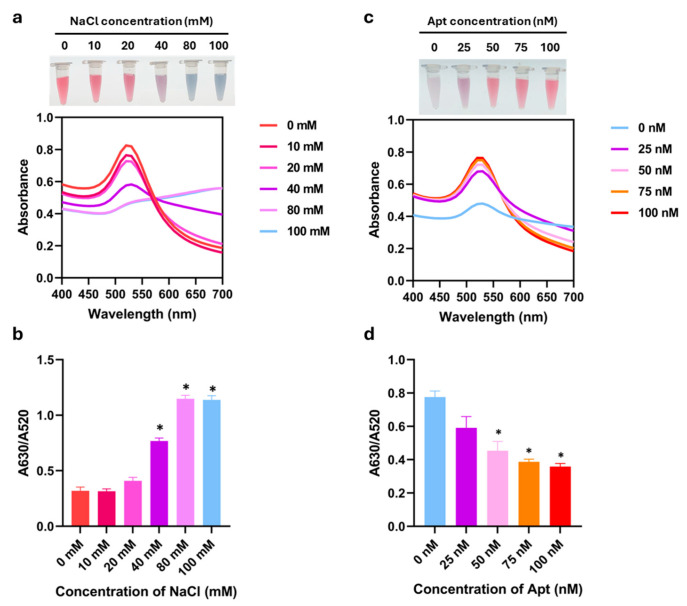
Optimization of NaCl and R8-su12 aptamer concentrations. UV-Vis absorption spectra with visual observation and absorbance peak ratio of A630/A520 nm of AuNPs with different concentrations of (**a**,**b**) NaCl and (**c**,**d**) R8-su12 Apt (Apt). All experiments were performed in three independent replicates, and results are presented as mean ± SEM (*n* = 3). * *p* < 0.05.

According to the colorimetric aptasensor system that relied on the salt-induced aggregation, the effect of NaCl concentration on the maximum absorption of AuNPs was investigated first. The absorption spectra of AuNPs under different concentrations of NaCl are shown in Figure 2a. Compared to the dispersed phase, the maximum absorption intensity at 520 nm decreased when NaCl concentration increased, indicating the aggregation of AuNPs. This aggregation resulted in an increased absorption peak at 630 nm that was consistent with the color change observed by the naked eye. The color changed from red to purple at 40 mM NaCl and turned gray when the NaCl concentration was up to 80 and 100 mM. At the NaCl concentration below 40 mM, the solution remained red. Correspondingly, the A630/A520 ratio significantly increased with the increase in the concentration of salts from 40 to 100 mM (Figure 2b). From these results, the minimal salt concentration that induced aggregation was considered to be 40 mM.

Subsequently, the optimal aptamer concentration was explored. In the absence of the R8-su12 Apt, the NaCl-AuNPs were purple-gray, and the UV-vis spectra revealed a weak absorption peak at 520 nm. This confirmed the aggregation state of AuNPs. As the concentration of the aptamer increased, the absorbance intensity of AuNPs at 520 nm increased, while the peak at 630 nm gradually disappeared (Figure 2c), indicating the more protective effects of the aptamer. As shown in Figure 2d, the strong protective signal was initially observed at the aptamer concentration of 50 nM, as indicated by the red color of the AuNP solution. Increasing the aptamer concentration over this point did not result in the color change, suggesting sufficient stabilization. This result was supported by the A630/A520 ratio, which demonstrated a significant decrease in aggregation starting at 50 nM (Figure 2d). Therefore, 50 nM was selected as the optimal aptamer concentration for the sensing system.

**Figure 3:**
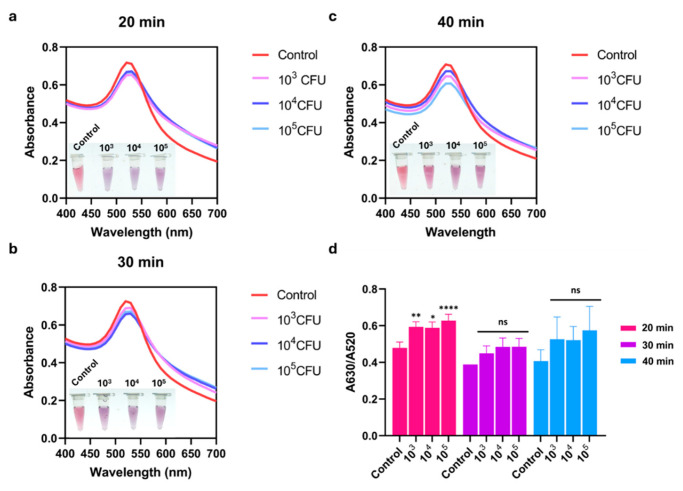
Optimization of binding time of R8-su12 Apt and *S. suis* S2 P1/7. (**a**) UV-Vis absorption spectra of AuNPs after (**a**) 20 min, (**b**) 30 min, and (**c**) 40 min reaction times between 50 nM R8-su12 Apt (Apt) and different numbers of *S. suis* S2 P1/7 (10^3^–10^5^ CFU). (**d**) Absorbance peak ratio of A630/A520 nm of AuNPs at different reaction time points. All experiments were performed in three independent replicates, and results are presented as mean ± SEM (*n* = 3). * *p* < 0.05, ** *p* < 0.01, **** *p* < 0.0001, and ns represents not significant.

The effect of reaction time between the aptamer and bacterial target was further investigated at 20, 30, and 40 min. As shown in Figure 3a–c, different incubation times had little influence on the detection system. The aggregate of AuNPs observed by the naked eye was also distinguishable from that of the control without the target. This visual change was correlated with the SPR spectra. The absorbance peak of AuNPs at 520 nm decreased in the presence of *S. suis* S2 P1/7, indicating target recognition and subsequent AuNP aggregation. Considering the A630/A520 ratio, the binding time at 20 min allowed a significant result, whereas extending the incubation to 30 and 40 min showed non-statistical differences compared to the control (Figure 3d). Therefore, the binding time of 20 min was selected as optimal with reduced assay time.

Based on the optimization results, the finalized colorimetric assay procedure was established. Briefly, the R8-su12 aptamer was thermally folded at 95 °C for 5 min and cooled at room temperature for 5 min to achieve a stable tertiary structure. In a colorimetric assay, 100 μL AuNPs were incubated with 10 μL of 50 nM aptamer for 15 min. Subsequently, 10 μL of bacterial suspension (or PBS for the blank) was added and incubated for 20 min to allow for target recognition and competitive displacement. Finally, a NaCl solution was added to reach a final concentration of 40 mM in a total reaction volume of 200 μL.

### 3.3. Specificity of the Colorimetric Aptasensor

**Figure 4:**
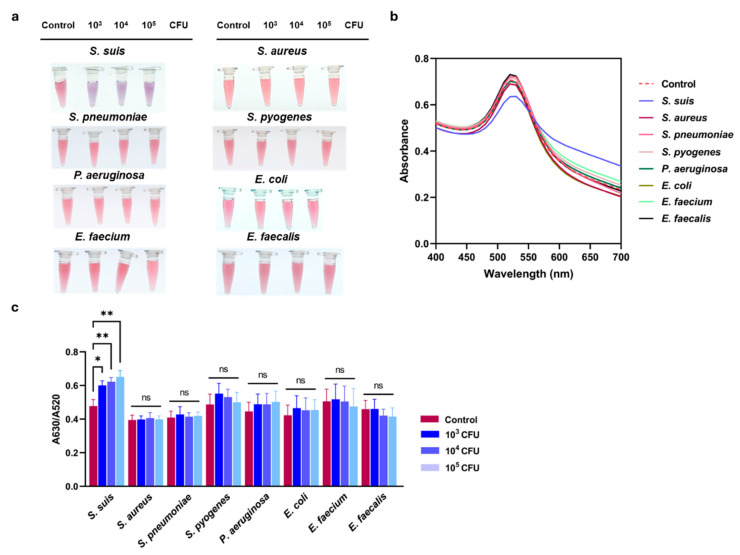
Specificity of colorimetric aptasensor. (**a**) Visual observation and (**b**) UV-Vis absorption spectrum of assay tested with other bacterial strains, including *S. aureus* ATCC 25923, *S. pneumoniae* ATCC 49619, *S. pyogenes* ATCC 19615, *P. aeruginosa* ATCC 27853, *E. coli* ATCC 25922, *E. faecium* ATCC 4743, and *E. faecalis* ATCC 4736, compared to *S. suis* S2 P1/7. (**c**) Absorbance peak ratio of A630/A520 nm analysis confirmed specificity of assay to *S. suis* S2 P1/7. All experiments were performed in three independent replicates, and results are presented as mean ± SEM (*n* = 3). * *p* < 0.05, ** *p* < 0.01, and ns represents not significant.

The specificity of the developed assay was further examined using other bacterial cultures, including *S. aureus* ATCC 25923, *S. pneumoniae* ATCC 49619, *S. pyogenes* ATCC 19615, *P. aeruginosa* ATCC 27853, *E. coli* ATCC 25922, *E. faecium* ATCC 4743, and *E. faecalis* ATCC 4736, compared to *S. suis* S2 P1/7. The result showed the red color of AuNPs that remained unchanged after the addition of other bacterial strains (Figure 4a), indicating the negative results of the reactions. Correspondingly, the SPR spectra and the A630/A520 ratio demonstrated no remarkable alteration (Figure 4b,c). This result suggested that the other bacteria did not react to the R8-Su12 Apt and confirmed the high specificity of this colorimetric assay.

### 3.4. Detection of S. suis Using the Colorimetric Sensing Method

**Figure 5:**
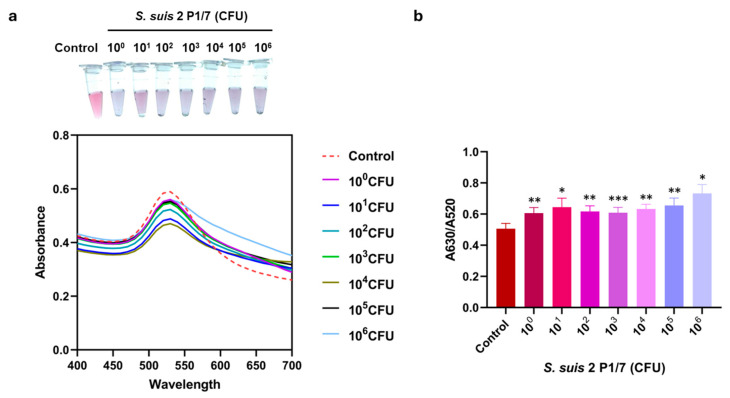
Determination of LOD of colorimetric aptasensor. LOD was evaluated through (**a**) UV-Vis absorption spectra with visual observation and (**b**) absorbance peak ratio of A630/A520 nm of assay with *S. suis* S2 P1/7 at various bacterial numbers (10^0^–10^6^ CFU). All experiments were performed in three independent replicates, and results are presented as mean ± SEM (*n* = 3). * *p* < 0.05, ** *p* < 0.01, and *** *p* < 0.001.

To evaluate the LOD of this assay, visual observation, SPR spectra, and the A630/A520 ratio were analyzed across a range of *S. suis* S2 P1/7 from 10^0^ to 10^6^ CFU. As shown in Figure 5, the color shift was observed by the naked eye starting at 1 CFU, which was consistent with the SPR spectral shift and a significant increase in the A630/A520 ratio compared to the control. These results indicated that the LOD of the colorimetric aptasensor was as low as 1 CFU of *S. suis*.

**Figure 6:**
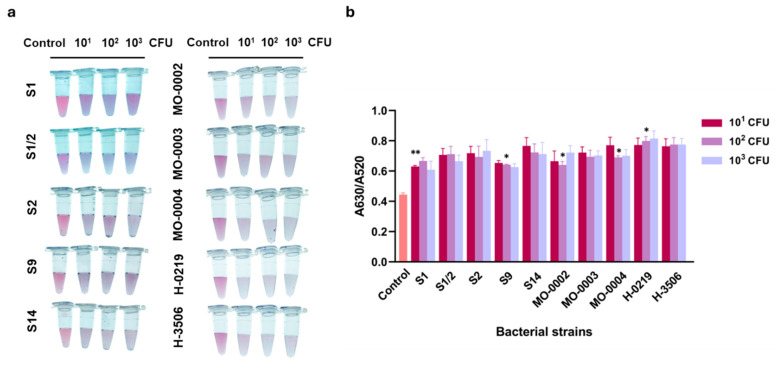
Broad serotype reactivity of colorimetric aptasensor with non-*S. suis* S2 P1/7 isolates. (**a**) Visual observation and (**b**) absorbance peak ratio of A630/A520 nm of assay with different *S. suis* serotypes (S1, S1/2, S2, S9, and S14) and *S. suis* clinical isolates (MO-0002, MO-0003, MO-0004, H-0219, and H-3506). All experiments were performed in three independent replicates, and results are presented as mean ± SEM (*n* = 3). * *p* < 0.05, ** *p* < 0.01.

Moreover, the colorimetric assay was assessed for its practicability for the detection of *S. suis*, which was not serotype S2 P1/7, including clinical isolates. As shown in Figure 6, the assay detected the *S. suis* S1, S1/2, S2, S9, and S14, and 5 positive samples of *S. suis*. This was evidenced by a significant increase in the A630/A520 ratio compared to the control. These findings suggested that the colorimetric aptasensor developed in this study exhibited broad serotype reactivity with various *S. suis* serotypes and could be performed reliably with the clinical isolates.

## 4. Discussion

*S. suis* infection is a zoonotic disease that significantly impacts veterinary and public health. Routine standard methods for identifying *S. suis* are time-consuming and can cause inconsistent findings. Despite the high precision of molecular techniques such as polymerase chain reaction (PCR) and enzyme-linked immunosorbent assay (ELISA), these require costly equipment along with complicated procedures, limiting their applicability in low-resource or field settings [10,11]. Biosensors serve as an alternative for rapid and reliable detection. These platforms can be designed based on different principles, including DNA-based, antibody-based, electrochemical, and colorimetric approaches. Several studies have introduced innovative biosensors for *S. suis* detection based on different principles. For instance, an electrochemical lateral flow immunoassay using the antibody against *S. suis* serotype 2 was developed [10], as was a loop-mediated isothermal amplification (LAMP)-based assay targeting the *S. suis* thrA housekeeping gene [12] and the glutamate dehydrogenase (gdh) gene [13]. These advances highlight the growing potential of biosensor technologies in facilitating timely and accessible diagnosis of *S. suis* infections.

In this study, we developed a label-free aptasensor based on AuNP colorimetric detection for simplified screening of *S. suis*. The assay utilized the unique optical properties of AuNPs synthesized by citrate reduction of HAuCl_4_. The negative charges of citrate anions are deposited on the AuNP surface, generating electrostatic repulsion that stabilizes the particles in aqueous dispersion. This dispersed state is characterized by the red color and the SPR spectrum absorption peak at 520 nm. Upon the addition of 40 mM NaCl, the electrostatic repulsion is reduced by the neutralizing effect of the Na^+^ ions [7]. As a result, the absorption peak intensity at 520 nm greatly declined while the intensity at 630 nm escalated, noticeable by a visible color change from red to purple. This characteristic indicates the aggregation state of AuNPs. Salt-induced aggregation protection occurs through the adsorption of aptamers onto citrate-capped AuNPs, which involves non-covalent electrostatic and ¶-stacking interactions, distributed along the nucleotide backbone [14,15]. From the result (Figure 2c,d), 50 nM was identified as the critical threshold that was sufficient to prevent salt-induced aggregation. By maintaining a lower surface coverage at this critical threshold, the aptamers are sufficiently spaced to undergo the conformational changes or displacement required to generate a detectable signal [16]. At this stage, the system is intentionally poised between stability and aggregation or near-critical (quasi-stable) conditions, a strategy commonly employed in high-sensitivity, label-free AuNP colorimetric sensors [16]. Under such conditions, even small perturbations in aptamer availability can destabilize the AuNPs and trigger aggregation, which is further amplified by the high extinction coefficient of AuNPs [16,17]. In addition, the aptamer may bind not only to bacterial cells but also to soluble target molecules released by the bacteria. Although the R8-su12 aptamer was selected against whole *S. suis*, it is likely to recognize surface-associated antigens or proteins, especially those involved in adhesion and biofilm formation, such as capsular polysaccharides, cell wall-associated proteins, and LPXTG-anchored surface proteins, because it can inhibit biofilm formation. Importantly, these molecules can be released or shed into the extracellular environment during bacterial growth. For example, muramidase-released protein (MRP) has been reported to be present in culture supernatants [18], and enolase has been detected in extracellular fractions in addition to the cell surface [19]. Therefore, even at very low bacterial concentrations (e.g., 1 CFU), the amount of released or shed target molecules may exceed the number of intact cells and contribute to aptamer binding. This provides an additional amplification mechanism that can enable detectable AuNP aggregation. Consistent with this interpretation, several ultrasensitive biosensors based on aptamers have reported detection limits in the range of ~1–5 CFU/mL [20,21], attributed to signal amplification and the detection of extracellular targets rather than strict one-to-one cell interactions.

Moreover, a key feature of the R8-su12 aptamer is 2′-F modification on all pyrimidine residues. These 2′-F groups alter the physicochemical properties of the RNA backbone. The high electronegativity of fluorine, compared to the native 2′-OH group, modifies the hydration shell and reduces the non-specific binding strength of the nucleobases to the gold surface [22]. This reduction in adsorption energy results in favorable desorption kinetics, allowing the aptamer to respond to the presence of *S. suis*. Utilizing R8-su12 Apt with AuNPs demonstrated the effective detection of *S. suis* S2 P1/7 through the visible color change of the aggregation state. A positive signal was observed within a binding time of 20–40 min, which is consistent with the reported reaction times in other aptamer-AuNP colorimetric assays. For example, the colorimetric assays employing AuNPs and a specific aptamer developed for *E. coli* O157:H7 [23], *Bacillus carboniphilus* [24], and *Salmonella typhimurium* [25] showed that effective detection was achieved in approximately 30 min, supporting the feasibility of this rapid detection for practical applications.

In terms of specificity, the assay also exhibited high specificity, showing no significant colorimetric response or aggregation when tested with non-target bacterial species, including *S. aureus*, *S. pneumoniae*, *S. pyogenes*, *P. aeruginosa*, *E. coli*, *E. faecium*, and *E. faecalis*. This obtained result is consistent with the previous evaluation of R8-su12 Apt specificity, which confirmed selective binding to *S. suis* over other bacterial species [4]. R8-su12 aptamer was selected by whole-cell SELEX against intact *S. suis* serotype 2 (P1/7) cells. The precise binding target has not yet been definitively identified by molecular characterization, which is a recognized limitation of whole-cell SELEX-derived aptamers. However, the broad serotype reactivity observed (S1, S1/2, S2, S9, S14) is consistent with the aptamer, recognizing the conserved surface epitope shared across *S. suis* serotypes. Structural genomics and biochemical studies of *S. suis* capsular polysaccharides and surface proteins suggest candidates such as conserved cell-wall proteins, lipoproteins, or non-type-specific polysaccharide backbone elements [26,27,28]. However, the specific target of R8-su12 should be required. Moreover, the developed assay demonstrated remarkable sensitivity, with an LOD as low as 1 CFU of *S. suis*, detected by a visual and spectrophotometric method. As summarized in Table 1, the sensitivity of the developed aptasensor is comparable to that of the LAMP-based assay, which also reported the detection limit at the lowest DNA content of 1 CFU of *S. suis* [13]. While an AuNP-based immunoassay using anti-*S. suis* capsular polysaccharide polyclonal antibodies reported a higher LOD of 1 × 10^4^ CFU [29], it is important to acknowledge that visual confirmation at 1 CFU is at the limit of naked-eye detection. Nevertheless, interpretation of the results requires trained and specialized personnel. The use of the A630/A520 absorbance ratio ensures the objective and quantitative validation of 1 CFU detection limit, removing the subjectivity associated with visual inspection. The improved sensitivity of the aptasensor assay was attributed to the high affinity and specificity of aptamer-target interactions.

Furthermore, broad serotype reactivity testing of the aptasensor assay with different *S. suis* serotypes showed positive detection for serotypes S1, S1/2, S2, S9, and S14, as well as clinical isolates. This suggested that the developed aptasensor had broad reactivity and recognized the conserved surface features shared across various *S. suis* serotypes [26,27,28], enhancing its applicability for broader screening tests and comprehensive surveillance. However, the closely related *Streptococcus* species such as *S. dysgalactiae*, *S. agalactiae*, *S. equi*, and *S. canis* should be tested in further study to strengthen the specificity of the developed aptasensor. As a result, this assay demonstrated potential as a rapid and simplified screening method for *S. suis*, enabling detection with either the naked eye or a UV-Vis spectrometer. The system exhibited high specificity without the need for extraction steps, specific probes, or antibodies. Furthermore, it operated independently of sophisticated equipment, highly trained personnel, or a lengthy analysis process. These advantages make it particularly suitable for application in both field and laboratory settings. Nevertheless, more *S. suis* from clinical samples should be validated to ensure the broad binding ability of the aptamer against *S. suis*. Testing should be done using actual clinical samples since they contain complex matrices that might seriously interfere with colorimetric assays [30,31]. Moreover, surface passivation techniques, such as polyethylene glycol (PEG) or blocking agents, would be required to enhance robustness for direct clinical deployment [32]. With further development, the assay could be effectively applied for routine monitoring and surveillance of *S. suis* over diverse samples, including clinical specimens, food products, and environmental sources.

## 5. Conclusions

In this study, we successfully developed a label-free AuNP-based colorimetric assay, utilizing R8-su12 Apt as an aptasensor for *S. suis* detection. The assay was based on the specific recognition of R8-su12 Apt and the optical properties of AuNPs as a colorimetric indicator, enabling rapid and visual detection by the naked eye. The aptasensor possessed high specificity toward *S. suis*, with no cross-reactivity observed among non-target bacterial species. Additionally, it exhibited sensitivity with an LOD of 1 CFU and had broad serotype applicability. With these benefits, the developed aptasensor demonstrates proof-of-concept feasibility and holds promise as a platform requiring further validation. This could be applicable as a potential platform for early screening and field surveillance of *S. suis* infection.

## Figures and Tables

**Table 1:** Comparative summary of detection methods for *S. suis*.

Detection Method	Target	LOD	Assay Time	Equipment	Sample Matrix	Ref.
Aptasensor (R8-su12/AuNPs)	Whole cell	1 CFU (spectrophotometry and visuality)	45 min	UV-Vis or naked eye	PBS, clinical isolates	This study
LAMP (*thrA* gene)	DNA	1 CFU equivalent	~60–90 min	Real-time PCR/heating block	Necropsy samples	[12]
LAMP (*gdh* gene)	DNA	1 CFU equivalent	~60–90 min	Real-time PCR/heating block	Raw pork	[13]
AuNP immunoassay	Capsular polysaccharide	1 × 10^4^ CFU	30 min	Spectrophotometer	Buffer	[29]
Electrochemical lateral flow	Serotype 2 antigen	Not specified	15–20 min	Portable potentiostat	Buffer	[10]
Traditional culture	Whole cell	10–100 CFU	18–48 h	Incubator, blood agar	Clinical specimens	Standard
PCR	Species-specific DNA	10–100 CFU	3–4 h	Thermal cycler	Clinical specimens	Standard

## Data Availability

The original contributions presented in this study are included in the article/Supplementary Materials. Further inquiries can be directed to the corresponding author.

## Supplementary Materials

The following supporting information can be downloaded at: https://www.mdpi.com/article/10.3390/bios16040215/s1, Figure S1: AuNPs synthesis and characterization. Table S1: Physicochemical characterization and batch-to-batch reproducibility of synthesized AuNPs (*n* = 2 independent batches). Table S2: Particle size, polydispersity index (PdI), and zeta potential (ZP) of synthesized AuNPs (*n* = 2 independent batches). Table S3: The UV-Vis spectrum of synthesized AuNPs over the range of 400–700 nm (*n* = 2 independent batches). Table S4: Bacterial strains used in this study. Table S5: Optimal experimental conditions for the developed aptasensor. Reference [33] is cited in the Supplementary Materials.

## Abbreviations

The following abbreviations are used in this manuscript:

Apt	Aptamer
AuNPs	Gold nanoparticles
SPR	Surface plasmon resonance
LOD	Limit of detection
CFU	Colony-forming unit
