# Supplementary material for: Aptamer-Based Label-Free Colorimetric Assay Using Gold Nanoparticles for Specific Detection of Streptococcus suis
Source: Biosensors (Basel). 2026 Apr 10;16(4):215. doi: 10.3390/bios16040215 (PMC13114957; doi:10.3390/bios16040215)
Supplement: Supplementary file 1 [file biosensors-16-00215-s001.zip › biosensors-4201050-supplementary.pdf]

biosensors

MDPI

Supporting Information

The physicochemical properties of the synthesized AuNPs were characterized across two independent batches. As shown in Figure S1a-d and Table S1, the particle size is approximately 19.39 ± 0.850 nm with a low polydispersity index (PdI) of 0.298 ± 0.004, indicating a uniform size distribution across separate syntheses. Moreover, the citrate capping provided a stable and negatively charged surface with ZP values of −37.27 ± 1.750 mV.

The UV-vis spectrum of AuNPs and STEM analysis (Figure S1e-f) confirmed the formation of monodispersed particles with a maximum surface plasmon resonance (SPR) peak at 520 nm, consistent with the particle size range of 15–20 nm [1]. Importantly, the A_{630}/A_{520} ratio, which is the primary signal for the colorimetric detection, was highly consistent at 0.299 ± 0.0098 across independent syntheses. This corresponds to an inter-batch relative standard deviation (RSD) of 3.28%, confirming that the starting material provides a uniform baseline for the assay.

From these results, the low variation across all critical parameters (RSD < 5%) indicates the reproducibility of the standardized synthesis method used in this study. The additional raw data were also provided in Table S2 and Table S3.

 https://doi.org/10.3390/bios16040215

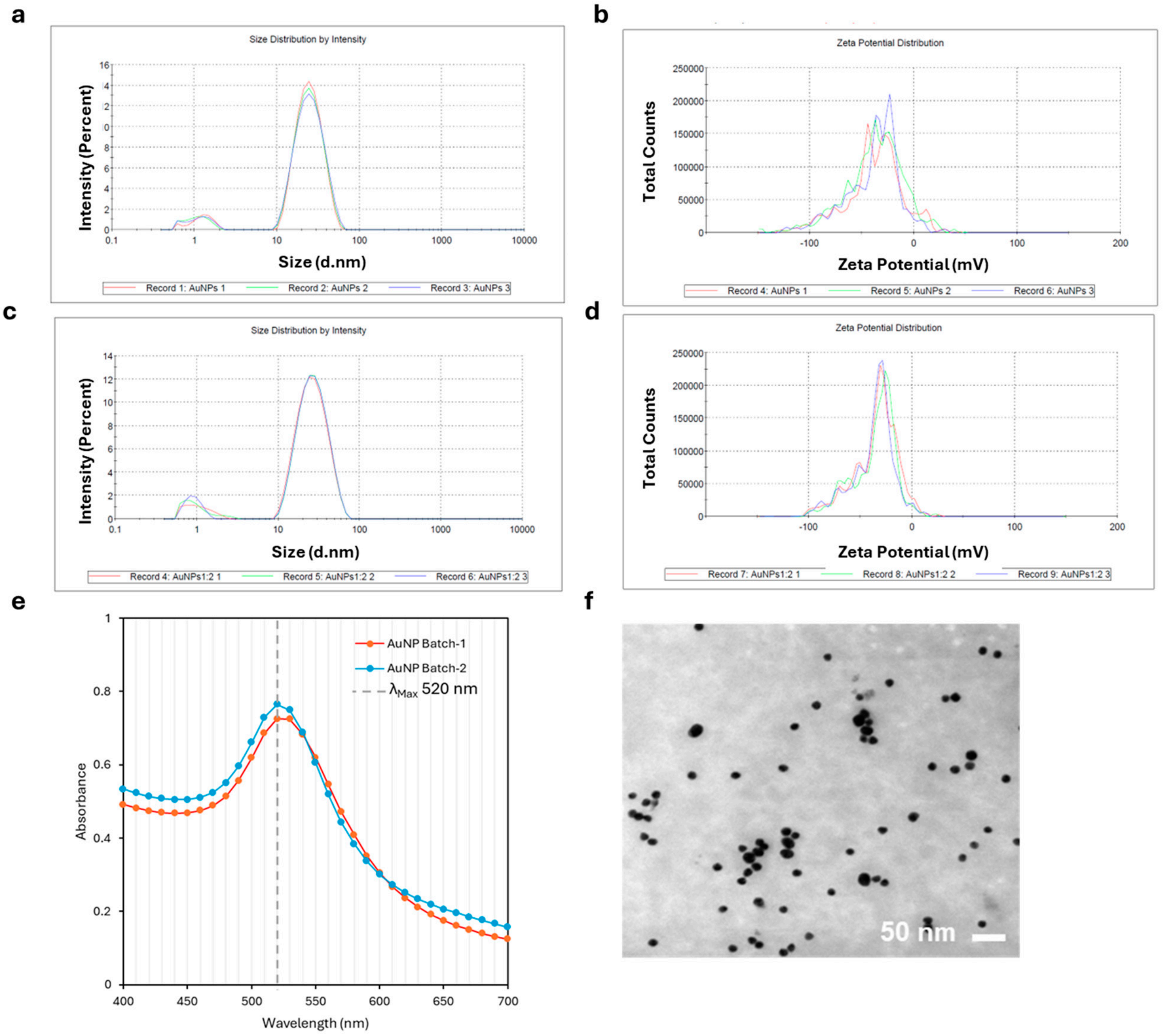

Figure S1. AuNPs synthesis and characterization. The hydrodynamic diameter of AuNPs with monodispersed characteristics and a negative surface charge of batch 1 (**a** and **b**), and batch 2 (**c** and **d**), respectively. The UV-Vis spectrum showed the absorbance profile of two independent batches of the synthesized AuNPs (**e**). The STEM image depicted the spherical morphology, confirming successful synthesis (**f**).

https://doi.org/10.3390/bios16040215

Table S1. Physicochemical characterization and batch-to-batch reproducibility of synthesized AuNPs (*n* = 2 independent batches).

Parameter	Batch 1 (n = 3)	Batch 2 (n = 3)	Average	% RSD (inter-batch)
Z-Ave (nm)	18.79 ± 0.030	19.99 ± 0.270	19.39 ± 0.850	4.38
Polydispersity index (PdI)	0.295 ± 0.004	0.301 ± 0.003	0.298 ± 0.004	1.42
ZP (mV)	−38.50 ± 0.960	−36.03 ± 1.370	−37.27 ± 1.750	4.69
A_{630}/A_{520} ratio	0.292	0.306	0.299 ± 0.0098	3.28

Table S2. Particle size, polydispersity index (PdI), and zeta potential (ZP) of synthesized AuNPs (*n* = 2 independent batches).

Scheme .	Batch 1			Batch 2		
	Z-Ave (d.nm)	PdI	ZP (mV)	Z-Ave (d.nm)	PdI	ZP (mV)
AuNPs 1	18.77	0.291	−38.1	19.70	0.302	-34.8
AuNPs 2	18.78	0.294	−37.8	20.04	0.304	-35.8
AuNPs 3	18.83	0.299	−39.6	20.24	0.298	-37.5
**Average**	**18.79**	**0.295**	**−38.5**	**19.99**	**0.301**	**-36.0**
**Std Dev**	**0.0322**	**0.004**	**0.964**	**0.2730**	**0.003**	**1.37**

Table S3. The UV-Vis spectrum of synthesized AuNPs over the range of 400–700 nm (*n* = 2 independent batches).

Wavelength (nm)	AuNP Batch-1	AuNP Batch-2
400	0.492	0.534
410	0.482	0.523
420	0.474	0.514
430	0.47	0.508
440	0.468	0.505
450	0.469	0.505
460	0.475	0.511
470	0.489	0.524
480	0.514	0.551
490	0.557	0.596
500	0.619	0.661
510	0.686	0.73
520	0.726	0.765
530	0.725	0.75
540	0.683	0.688
550	0.62	0.606
560	0.546	0.52
570	0.473	0.444
580	0.409	0.384
590	0.352	0.337
600	0.306	0.301
610	0.267	0.273
620	0.237	0.251
630	0.212	0.234
640	0.192	0.219
650	0.175	0.206
660	0.161	0.196
670	0.15	0.185
680	0.14	0.176
690	0.131	0.167
700	0.124	0.157

https://doi.org/10.3390/bios16040215

Table S4. Bacterial strains used in this study.

Category	Strain/Serotype	Source/Collection	Purpose
**Target organism**	*S. suis* serotype 2 (P1/7)	Reference strain	Optimization & LOD determination
**Serotype panel**	*S. suis* S1	Clinical isolates	Broad serotype reactivity testing
	S. suis S1/2	Clinical isolates	Broad serotype reactivity testing
	S. suis S2	Clinical isolates	Broad serotype reactivity testing
	S. suis S9	Clinical isolates	Broad serotype reactivity testing
	S. suis S14	Clinical isolates	Broad serotype reactivity testing
**Clinical validation**	Clinical *S. suis* isolates (n = 5)	Patient specimens	Proof of concept for real-sample applicability
**Specificity panel**	*S. aureus* ATCC 25923	ATCC reference	Cross-reactivity testing
	S. pneumoniae ATCC 49619	ATCC reference	Cross-reactivity testing
	S. pyogenes ATCC 19615	ATCC reference	Cross-reactivity testing
	P. aeruginosa ATCC 27853	ATCC reference	Cross-reactivity testing
	E. coli ATCC 25922	ATCC reference	Cross-reactivity testing
	E. faecium ATCC 4743	ATCC reference	Cross-reactivity testing
	E. faecalis ATCC 4736	ATCC reference	Cross-reactivity testing

Table S5. Optimal experimental conditions for the developed aptasensor.

Parameter	Optimized Value	Range Tested	Selection Rationale
AuNP incubation	15 min	-	Aptamer adsorption equilibration
R8-su12 aptamer	50 nM	0–100 nM	Optimal protection against salt-induced aggregation
Binding time	20 min	20–40 min	Significant detection with minimal assay time
NaCl concentration	40 mM	0–100 mM	Minimum concentration for AuNP aggregation
Detection wavelengths	520 nm, 630 nm	400–700 nm	SPR peak (dispersed) vs aggregation indicator
Signal measurement	A_{630}/A_{520} ratio	-	Quantitative aggregation assessment
Total assay time	45 min	-	Including all incubation steps
Detection range	10^{0}–10^{6} CFU	-	Dynamic range of assay

Reference

1. Tirkey, A.; Babu, P.J. Synthesis and characterization of citrate-capped gold nanoparticles and their application in selective detection of creatinine (A kidney biomarker). *Sens Int* **2024**, *5*, 100252.

https://doi.org/10.3390/bios16040215
